# How Bees Respond Differently to Field Margins of Shrubby and Herbaceous Plants in Intensive Agricultural Crops of the Mediterranean Area

**DOI:** 10.3390/insects11010026

**Published:** 2019-12-29

**Authors:** Juan Antonio Sanchez, Aline Carrasco, Michelangelo La Spina, María Pérez-Marcos, F. Javier Ortiz-Sánchez

**Affiliations:** 1 Biological Control & Ecosystem Services Laboratory, Instituto Murciano de Investigación y Desarrollo Agrario y Alimentario (IMIDA), C/Mayor s/n, E-30150 La Alberca, Spain; carrasco.alina@gmail.com (A.C.); laspinamichelangelo@gmail.com (M.L.S.); mriaperez@gmail.com (M.P.-M.); 2 Grupo de Investigación “Transferencia de I+D en el Área de Recursos Naturales”, Universidad de Almería, Ctra, de Sacramento s/n, E-04120 La Cañada de San Urbano, Spain; fjortiz@ual.es

**Keywords:** pollinating insects, bees, floral edges, biodiversity, agroecosystems, conservation

## Abstract

(1) Intensive agriculture has a high impact on pollinating insects, and conservation strategies targeting agricultural landscapes may greatly contribute to their maintenance. The aim of this work was to quantify the effect that the vegetation of crop margins, with either herbaceous or shrubby plants, had on the abundance and diversity of bees in comparison to non-restored margins. (2) The work was carried out in an area of intensive agriculture in southern Spain. Bees were monitored visually and using pan traps, and floral resources were quantified in crop margins for two years. (3) An increase in the abundance and diversity of wild bees in restored margins was registered, compared to non-restored margins. Significant differences in the structure of bee communities were found between shrubby and herbaceous margins. *Apis mellifera* and mining bees were found to be more polylectic than wild Apidae and Megachilidae. The abundance of *A. mellifera* and mining bees was correlated to the total floral resources, in particular, to those offered by the Boraginaceae and Brassicaceae; wild Apidae and Megachilidae were associated with the Lamiaceae. (4) This work emphasises the importance of floral diversity and shrubby plants for the maintenance of rich bee communities in Mediterranean agricultural landscapes.

## 1. Introduction

Agriculture and other human activities have greatly transformed the natural landscapes in big extensions of the Earth’s surface [1]. The most evident impact of modern agriculture is the tremendous reduction of biodiversity, with the substitution of heterogeneous agricultural landscapes by homogeneous stands of cultivated plants managed in a very intense way [2,3]. This drastic environmental transformation has had a great impact on pollinating insects, wild bees being one of the animal groups which suffered more severely from the intensification of agriculture [4,5,6,7,8,9,10,11]. Bees are especially abundant in Mediterranean ecosystems, where they may represent more than 90% of the pollinating insects [12,13,14]. Several aspects of agricultural intensification influence negatively pollinating insects, but habitat loss and fragmentation are considered as the principal ones [10,15,16,17,18,19,20,21,22,23]. The intensification of agriculture has led to increasing plot size and to the elimination of edges that provided floral resources and nesting sites [4,24,25,26,27,28,29,30]. Besides, the continuous use of herbicides in conventional farms keeps crops free from ruderal plants, reducing the availability of nectar and pollen [2,5,6,7,8,10,31,32,33,34]. In addition, some of the chemicals used for pest control cause direct mortalities and have sublethal effects on pollinating insects [35,36,37,38,39]. Modern intensive agriculture creates a paradoxical scenario because, on the one hand, it tries to maximize yield to the extreme, and, on the other, it adopts practices that eliminate the pollinating insects upon which the production of many crops depends. It is estimated that 35% of the global crop production depends on pollinators [25,40]; among these, bees are the most important group, providing essential services to both wild and cultivated plants [25,41,42].

Bees (Apoidea, Anthophila) suffer particularly the impact of the degradation of agricultural landscapes because of their strict dependence on diverse floral resources [25,42]. A decline in bee populations, both wild and managed, has been reported worldwide [6,7,10,11,19,43,44,45,46,47]. About nine percent of the European bees are known to be threatened, but it is quite likely that the true figure is greater because of the many species with a “Data Deficient” status [48]. The drastic decline in bee populations has generated worldwide alarm, both in the general public and in the scientific community [7,10]. Some measures have been taken regarding the regulation of certain insecticides, such as neonicotinoids, in some parts of the world (i.e., Europe) [49], but the problem of habitat loss remains largely unsolved. Several authors have investigated the management of vegetation to restore the functional diversity lost through the intensification of agriculture [23,50,51,52,53,54]. The sowing of floral strips along crop margins is a strategy commonly used for the restoration of habitat in farmlands [55]. Floral margins can provide both floral resources and suitable nesting sites for pollinating insects [56,57,58,59]. Edges of wild vegetation may be critical for the maintenance of the communities of pollinating insects in an agricultural landscape with low representation of natural vegetation or in the absence of crops offering a continuous floral resource [60]. Several studies have shown that edges with varied flowering plants increase the abundance and diversity of wild and managed pollinating insects [29,57,61,62,63,64,65,66,67,68,69,70,71,72,73,74,75,76]. However, with the exception of bumble bees, just a few studies have evaluated the effect of floral margins on the abundance and diversity of wild bees [29,77,78].

Because of the polylectic habits and disparate needs of the different bee species, together with the short blossoming period and variation in the floral structure of different plant species, plant diversity is vital for the maintenance of bee communities [14,27,55,57,79,80]. Many bee species are known to adopt a generalist strategy but in some cases, pollinators and plants are linked by specific floral traits [81,82]. However, very little is known about the structure of bee communities in relation to the variation in plant assemblages. Most of the works on the restoration of field margins have been carried out using herbaceous plant species in temperate regions, while practically no consideration has been given to the use of shrubby species for the creation of permanent structures in the Mediterranean area. The natural vegetation in the Mediterranean area includes a great diversity of shrubs pollinated by many insect species, particularly bees [13,42,83,84,85]. Therefore, schemes aimed at the conservation of pollinating insects should necessarily take into consideration shrubby plants.

The aim of this work was to determine how planting shrubs and herbs may contribute to the increase of the abundance and diversity of bees in the surroundings of crops in areas of intensive agriculture. It was hypothesised that vegetated crop margins would show a higher abundance and diversity of bees than non-restored margins and, because of their differing floral compositions, herbaceous and shrubby margins would differ qualitatively and quantitatively on the bees that visit them. The work was focused on bees because they represent the most important group of pollinating insects in Mediterranean areas [12,13,14,83,84,85].

## 2. Materials and Methods

### 2.1. Design and Setting of the Experiment

The assay was carried out during 2011 and 2012 in a farm near the locality of Pulpí (Almería) in southern Spain (37°18′ N, 1°46′ W). In the vicinity of the farm, there were olive and citrus orchards, vegetable crops, e.g., lettuce, and small extensions of the natural vegetation of the garrigue type composed by a high diversity of shrubby plants from the Lamiaceae family, such as thyme and rosemary, Cistaceae, and Fabaceae, such as genista. The community of bees was studied in three types of margins bordering intensively managed spinach crops: (1) margins vegetated with shrubby plants composed mainly of Lamiaceae species (Table 1); (2) margins sown with a mixture of herbaceous plants belonging to several families (Table 1); (3) margins with no vegetation. The spinach crops were managed in a conventional way for the control of insect pests and fungal diseases. The margin strips were 25 m long and 3 m wide (75 m^2^). Each of the three margin types was replicated twice and assigned randomly to each of the two spinach fields. The separation between the different margin strips within the same spinach field was at least 20 m and the distance between the two spinach fields was 1.5 km. The herbaceous seed mixture (2.1 g/m^2^) was sown manually in the autumn of 2010 (Table 1). The herbaceous margin was mowed in the late summer of 2011 and was left to regrow. Shrubs were transplanted in the year prior to the experiment, in mid-January 2010 (Table 1). For the two types of margins, plants were selected to provide continuous blossoming from spring to early summer. The vegetated plots were irrigated once every one or two weeks, and the shrubby margins were weeded periodically. Non-vegetated margins were kept free from vegetation by the manual removal of plants and the use of herbicides.

### 2.2. Sampling of Bees and Vegetation

The abundance of bees in floral margins was estimated by visual and pan trap sampling. Because of the absence of vegetation in control margins, visual sampling was performed only in shrubby and herbaceous margins. Visual sampling was carried out by counting the number of bees within a 2 × 2 m square during a 4-min period. Each square was sampled by two scouts. Due to the difficulties in identifying bees by visual samplings, bees were grouped into four categories: (1) *Apis mellifera*, (2) wild Apidae, (3) Megachilidae and (4) mining bees; this latter group included Andrenidae, Halictidae, Colletidae and Melittidae. The number of each group of bees visiting flowers was registered independently for the different plant species within the sampling square. The sampling procedure was repeated three times for each of the two replicates of the shrubby and herbaceous margins on each date. Bees were sampled on six and eight dates during the first and second years, respectively. Sampling was performed in sunny conditions with a temperature above 20 °C and a low wind speed. The yellow pan traps were 28 cm in diameter and 14 cm high and were filled with water, formaldehyde (1%) and a drop of detergent [24]. The yellow colour was used because it was known to collect the highest richness of bee species [86]. Three pan traps were used per strip. The traps were emptied every two weeks. The specimens were preserved in 70% alcohol until they were dried and mounted for their identification. They were identified to the species level whenever possible. However, some were identified just to the genus or family level, either because they were not in good shape and the characters used for the identification were not visible or because of the lack of reliable taxonomic keys or specimens of reference. The reference collection of voucher specimens is held by the Instituto Murciano de Investigación y Desarrollo Agrario y Alimentario (IMIDA). Visual and pan trap samplings were carried out from mid-April (2011) or March (2012) to the beginning of July. The visual samplings of weeks 19, 21 and 22 in the first year and of week 27 in the second year could not be carried out because of the bad weather conditions (e.g., strong winds). The cover and blossoming of each plant species were estimated in the same 2 × 2 m squares in which bees were sampled. The percentage of blossoming plants was estimated by inspecting 20 plants per species and counting the number of them in bloom. The floral resources offered by a given plant species within the sampling square were estimated by multiplying the proportion of its cover by the proportion of plants in bloom.

### 2.3. Analysis of Data

*Analyses of bee diversity*. Generalised mixed effect models (GLMMs), with the function “glmmPQL” (“MASS” package) set to the Gaussian distribution with the link “log” [87], were used to determine the effect of margin type (fixed factor) on the richness of bee genera and species, using the data from the pan trap sampling. The captures of the three pan traps of each replicate were averaged for each sampling date. The year was introduced in the models as a fixed factor and date of sampling as a random one. The contrast among margin types was tested using “Tukey” with the function “glht” in the “multcomp” package [88]. The same procedure was used to compare the Shannon-Wiener diversity index among margin types. More details about the models and procedures are given as Supplementary Material (File 1). This index was calculated using the abundance of species in pan traps, with the “diversity” function in the “vegan” package [89].

*Bee abundance in margin types*. GLMMs, using the function “glmmPQL” set to the Gaussian distribution with the link “log”, were also used to test the effects of the margin type and year, as fixed factors, on the abundance of *A. mellifera* and wild bees, separately, on each sampling date (random factor) in pan traps. The same models were used to test for specific differences within the various groups of wild bees individually (i.e., wild Apidae, Andrenidae, Halictidae and Megachilidae). In this case, because of their low abundances, monthly averages of the captures on the pan traps were used and, thus, “month” was introduced in the models as a random factor (Supplementary Material, File 2). The contrast among margin types was performed in the same way as in the previous analysis. The same approach was used to test for the effect of margin type (i.e., herbaceous or shrubby) and year on the number of the different group of bees (i.e., *A. mellifera,* wild Apidae, Megachilidae and mining bees) registered in the visual samplings (Supplementary Material, File 3). The numbers of each group of bees registered in the three 2 × 2 m squares sampled in each of the two replicates were averaged for each sampling date. Besides, the data of the same month were averaged for the analyses.

*Structure of bee communities and floral resources*. Differences in the community of bees that visited all the plant families were tested by PERMANOVA, using the function “adonis”, and by Bray-Curtis distances calculated with the “vegdist” function, both functions being available in the “vegan” package [89]. Kruskal’s non-metric multidimensional scaling (NMDS), using the Bray-Curtis distance and *K* = 3, was applied to find out how plant families clustered in relation to the groups of bees that visited them. The function “metaMDS” in the “vegan” package was used to perform NMDS on the number of the four groups of bees registered on each plant family within the 2 × 2 m sampling squares on each sampling date (Supplementary Material, File 4). The relationship between the abundance of the different groups of bees in the visual sampling and the total floral resources (i.e., the sum of the floral resources of all the plant families) or the floral resources of each plant family (i.e., Apiaceae, Asteraceae, Boraginaceae, Brassicaceae, Fabaceae and Lamiaceae), introduced individually as fixed factors, was tested using the “glmmPQL” function (“MASS” package) (Supplementary Material, File 5). For *A. mellifera* and mining bees, the data fit the log-normal function, thus, the family was set to the Gaussian distribution with the link “log”, while in the case of the Megachilidae, the function was set to the negative binomial family. The data of the wild Apidae did not fit any of the most common distributions; thus, the relationship between bee abundance and floral resources was not tested. GLMMs, set to the Gaussian distribution with the link “log”, were also used to test the effect of the floral resources on the number of genera and species, and on the Shannon-Wiener diversity index (Supplementary Material, File 6).

## 3. Results

### 3.1. Diversity of Bees

**Figure 1:**
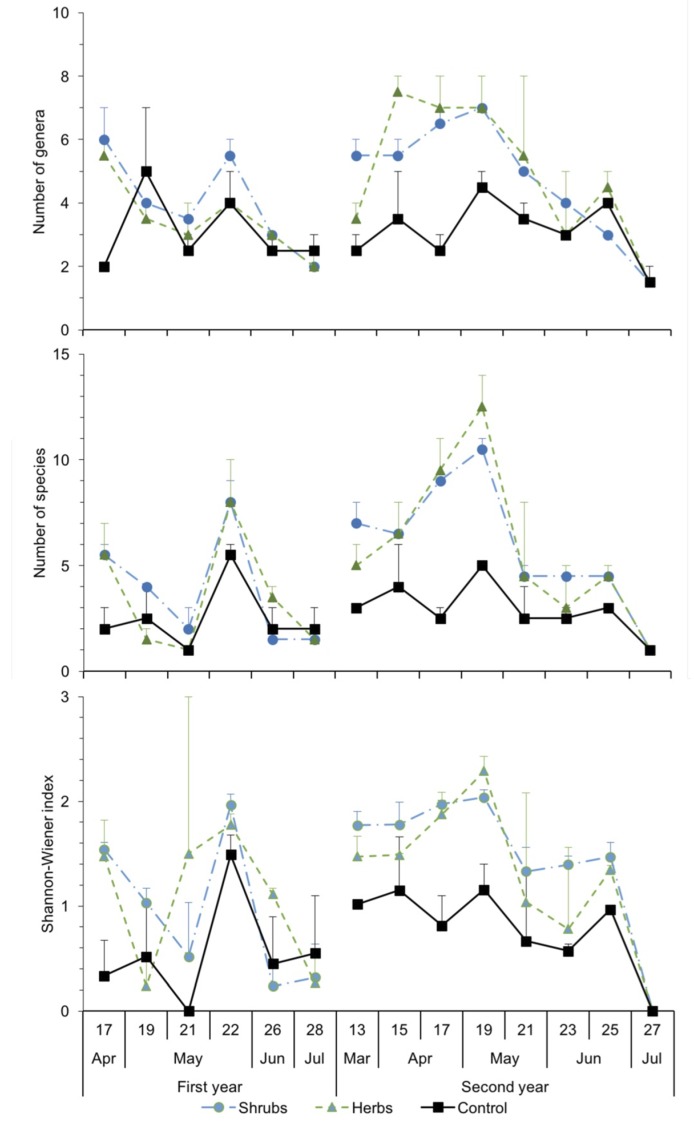
Numbers of genera (upper chart) per margin type on each sampling date and species (central) and the Shannon-Wiener index (lower) in pan traps in shrubby, herbaceous and control margins (means±SE). The numbers in the abscise (x) axe indicate the week of the year.

Over the two years of the study, 2374 bees captured in pan traps were identified to the family, 2141 to the genus and 1562 to the species level. *Apis mellifera* (35.3%) together with the genera *Eucera* (24.9%), *Andrena* (17.5%), *Lasioglossum* (14.2%) and *Panurgus* (2.7%) represented about 95% of the specimens captured in pan traps. Other minor genera were *Anthidium, Ceratina, Colletes, Halictus, Hoplitis, Hylaeus, Melitta, Megachile, Nomioides, Nomada, Osmia, Rhodanthidium* and *Sphecodes* (Table 2). The richness of genera in shrubby and herbaceous margins was significantly higher than in non-vegetated margins (x^2^ = 22.8, df = 2, *p* < 0.001; Tukey contrast, *p* < 0.001) (Figure 1), but no significant differences were found between shrubby and herbaceous margins (Tukey contrast, *p* > 0.05). In the first year, the number of genera peaked twice, once in mid-April and again at the end of May; in the second year, the number of genera increased progressively to reach a maximum by mid-May (Figure 1). In shrubby and herbaceous margins, the number of genera increased between the first and the second year (Figure 1), but the differences between years were not statistically significant (x^2^ = 1.23, df = 1, *p* = 0.266).

A total of 58 species were identified from the samples collected in pan traps. *Apis mellifera*, *Eucera notata*, *Lasioglossum interruptum*, *Andrena flavipes, Lasioglossum malachurum* and *Panurgus cephalotes* were the most common species (Table 2). Two of the species captured in shrubby and herbaceous margins, *Lasioglossum mandibulare* and *Andrena ovatula*, are catalogued as near threatened. Forty-seven species were collected very occasionally, representing less than 1% of the total of the individuals identified to the species level (Table 2). Ten of these species are catalogued as “Data Deficient” in the red book of bees, including *Andrena lepida* Schenk, *Andrena nilotica* Warncke, *Andrena thoracica* (Fabricius), *Anthidium taeniatum* Latreille and *Rhodanthidium sticticum* (Fabricius). Many of the species (38 out of 45) that are considered as of “Less Concern” in the red book were collected very occasionally (<1%).

The dynamics of the number of species followed a trend similar to that of the genera (Figure 1). In the two years of the study, the average number of species peaked around mid-May; an increase in the number of species was registered between the first and second years but the difference was not statistically significant (x^2^ = 2.16, df = 1, *p* = 0.141). As in the case of the bee genera, the number of species in shrubby and herbaceous margins (40 and 43 species, respectively) was significantly higher than in non-vegetated margins (26 species) (x^2^ = 45.9, df = 2, *p* < 0.001; Tukey contrast, *p* < 0.001) (Figure 1), but no significant differences were found between shrubby and herbaceous margins (Tukey contrast, *p* > 0.05). Significant differences in the Shannon-Wiener diversity index were found between vegetated and non-vegetated margins (x^2^ = 22.6, df = 2, *p* < 0.001; Tukey contrast, *p* < 0.001), but not between shrubby and herbaceous margins (Tukey contrast, *p* > 0.05) (Figure 1). The average diversity index peaked concomitantly with the number of species, around mid-May, with a notable but not significant increase in diversity between the first and the second year (x^2^ = 2.16, df = 1, *p* = 0.142) (Figure 1). More details about the models, procedures and results of these analyses are given as Supplementary Material (File 1).

### 3.2. Abundance and Dynamics of Bees in Margins: Pan Traps and Visual Samplings

**Figure 2:**
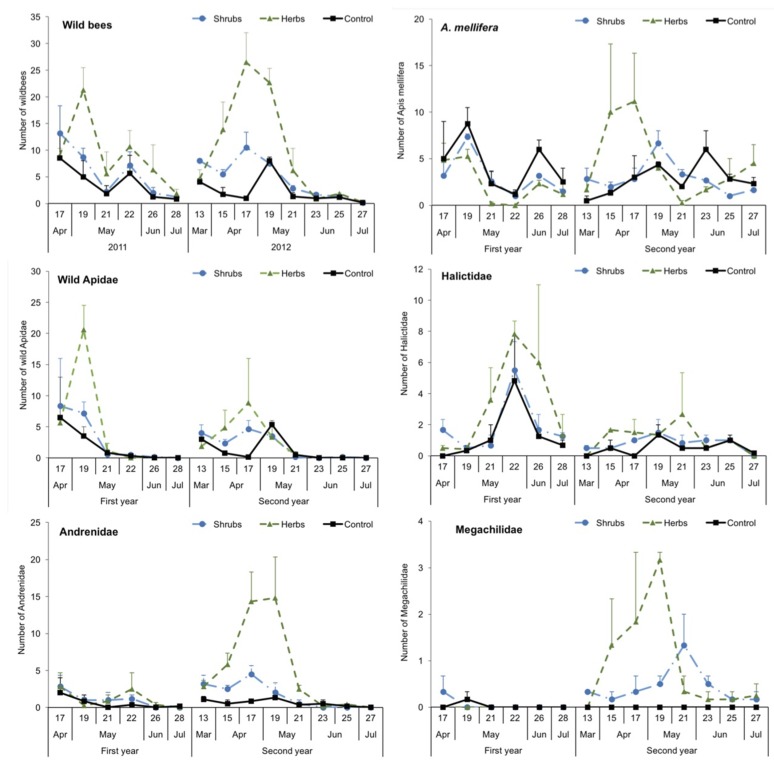
Trends in the numbers of wild bees (means ± SE), *A. mellifera*, wild Apidae, Halictidae, Andrenidae and Megachilidae captured in pan traps in shrubby, herbaceous and control margins. The numbers in the abscise (x) axe indicate the week of the year.

The capture of *A. mellifera* in pan traps peaked twice between April and June (Figure 2). Wild bees peaked generally in April-May and experienced a great reduction in their abundances from June onwards. The variation in the number of captures of *A. mellifera* was similar for the three kinds of margins (x^2^ = 1.43, df = 2, *p* = 0.489) (Figure 2). In contrast, the type of margin had a significant effect on the captures of wild bees (x^2^ = 65.2, df = 2, *p* < 0.001), the number of wild bees being significantly higher in shrubby and herbaceous than in control margins (Tukey contrast, *p* < 0.05) (Figure 2), and in herbaceous margins compared to shrubby margins (Tukey contrast, *p* < 0.001; Supplementary Material, File 2). Qualitative differences among the margin types were observed when the abundance of bees was analysed at the family level. In the case of wild Apidae, no significant differences were found among the different margins (x^2^ = 3.70, df = 2, *p* = 0.158) but the number of captures was significantly higher in the first than in the second year (x^2^ = 7.48, df = 1, *p* = 0.006). The captures of Halictidae were influenced by the margin type (x^2^ = 29.5, df = 2, *p* < 0.001), being significantly higher in herbaceous than in shrubby and control margins (Tukey contrast, *p* < 0.001), but similar between shrubby and control margins (Tukey contrast, *p* = 0.474) (Figure 2) (Supplementary Material, File 2).

The captures of Halictidae were significantly higher in the first than in the second year (x^2^ = 20.9, df = 1, *p* < 0.001). The Andrenidae were also highly influenced by the type of margin (x^2^ = 52.6, df = 2, *p* < 0.001) and year (x^2^ = 27.6, df = 2, *p* < 0.001), their captures being higher in herbaceous than in shrubby and control margins in the second year but not in the first (interaction, x^2^ = 13.6, df = 2, *p* = 0.001) (Tukey contrast, *p* < 0.001; Supplementary Material, File 2) (Figure 2). No significant differences were found in the number of Megachilidae captured in relation to the type of margin (x^2^ = 1.25, df = 2, *p* = 0.535).

**Figure 3:**
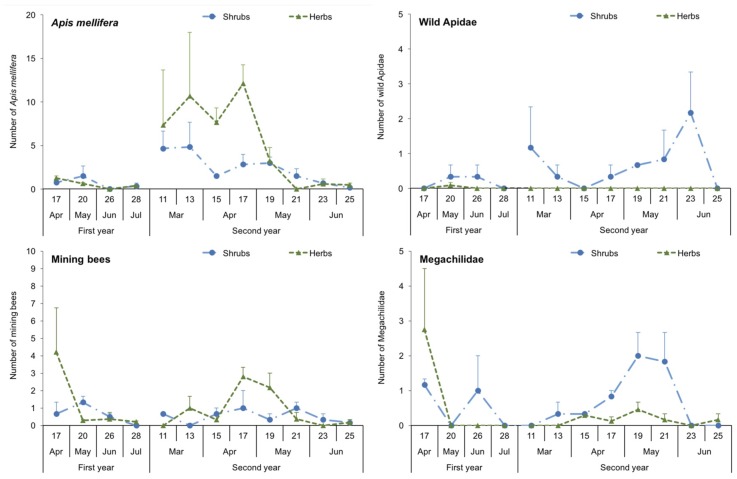
Visual sampling. Numbers of *A. mellifera*, wild Apidae, mining bees and Megachilidae observed in 2 × 2 m squares over a four-minute period in shrubby, herbaceous and control margins (means±SE). The numbers in the abscise (x) axe indicate the week of the year.

In several groups of bees, the estimation of bee abundances differed between the pan traps and the visual sampling (Figure 2 and Figure 3). For instance, a significantly higher number of *A. mellifera* was registered in herbaceous than in shrubby margins (x^2^ = 29.4, df = 1, *p* < 0.001), and in the second than in the first year (x^2^ = 12.5, df = 1, *p* < 0.001). Mining bees showed a response similar to that of *A. mellifera*, with differences between margins (x^2^ = 7.68, df = 1, *p* = 0.006) and years (x^2^ = 6.30, df = 1, *p* = 0.012). In contrast, the number of wild Apidae was significantly higher in shrubby than in herbaceous margins (x^2^ = 8.61, df = 1, *p* = 0.003), and in the second than in the first year (x^2^ = 156.4, df = 1, *p* < 0.001). In the case of the Megachilidae, no differences were found neither for margin type (x^2^ = 1.23, df = 1, *p* = 0.267) nor for year (x^2^ = 0.397, df = 1, *p* = 0.529). For more details about these statistical analyses see the Supplementary Material, File 3.

### 3.3. Structure of Bee Communities and Floral Resources

**Figure 4:**
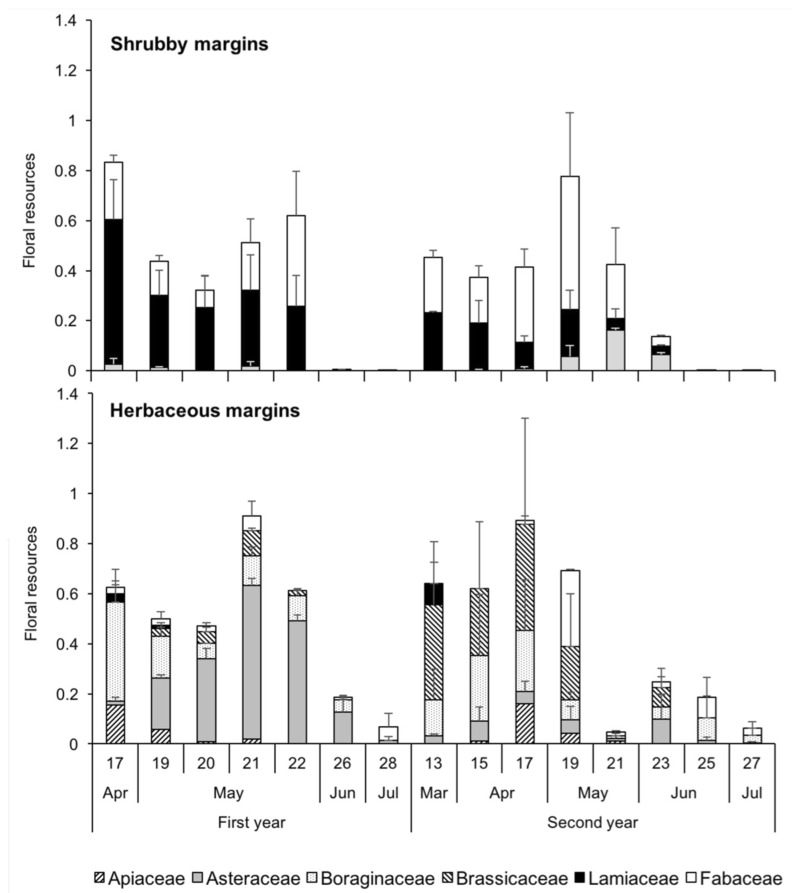
Trends in the floral resources (i.e., the proportion of cover*proportion of the plant in bloom) of different plant families in shrubby (upper) and herbaceous (lower) margins (means±SE). The numbers in the abscise (x) axe indicate the week of the year.

Plant species of the Lamiaceae and Fabaceae (Table 1) represented most of the floral resources in shrubby margins (Figure 4). Plants of these families blossomed mainly in the Spring of the first and second years. Asteraceae were infrequent in shrubby margins and blossomed in late Spring, especially in the second year. In herbaceous margins, Boraginaceae and Asteraceae offered most of the floral resources in the first year: *Borago officinalis* L. and *Echium vulgare* L. blossomed abundantly in mid-Spring and were replaced by *Calendula officinalis* L. from mid-May onwards (Figure 4). In the second year, the floral resources were similar to those of the first, with the exception that Asteraceae were uncommon and almost completely replaced by Brassicaceae.

**Figure 5:**
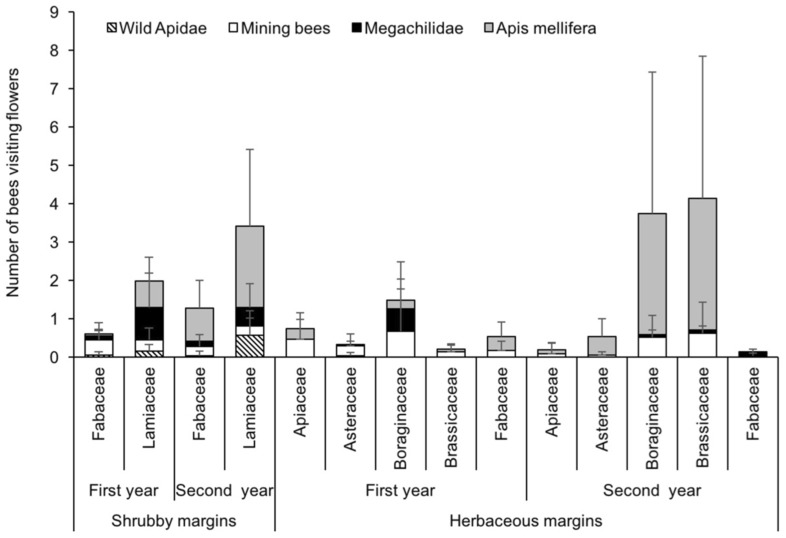
Annual averages of the different bee groups (*A. mellifera*, wild Apidae, mining bees and Megachilidae) observed in 2 × 2 m squares over a four-minute period in shrubby, herbaceous and control margins (means ± SE).

**Figure 6:**
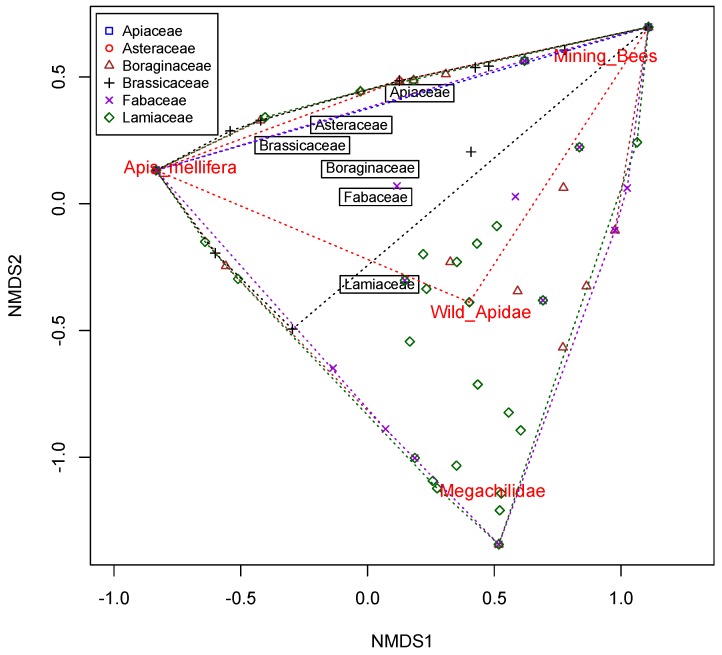
Plot of the scores of the first two components for the different plant families (symbols) and bee groups (red labels) in the of non-metric multidimensional scaling (NMDS). The black labels indicate the centroids for the different plant families.

*Apis mellifera* and mining bees were registered on the six plant families, Megachilidae on four (Boraginaceae, Brassicaceae, Fabaceae and Lamiaceae) and wild Apidae on three (Asteraceae, Fabaceae and Lamiaceae) (Figure 5). Of the 19 plant species available in vegetated margins, *A. mellifera* was registered on 17, mining bees on 14, Megachilidae on 12 and wild Apidae on five. In herbaceous margins, *A. mellifera* was mainly observed on *Echium vulgare* (43.2% of the total *A. mellifera* observed in herbaceous margins) and *Diplotaxis catholica* (25%), while in perennial margins it was mainly observed on *Ballota hirsuta* (20.6% of its total in shrubby margins)*, Lavandula dentata* (22.2%) and *Lavandula stoechas* (26.1%). Mining bees were mainly observed on herbaceous plants *E. vulgare* (43.2%) and *D. catholica* (25.0%), wild Apidae on perennials *B. hirsuta* (65.8%) and *L. dentata* (26.3%) and Megachilidae on both herbaceous *E. vulgare* (65.5%) and shrubby *B. hirsuta* (65.6%) plants. The PERMANOVA showed a significant effect of the plant families on the abundance of the different bee species in herbaceous and shrubby margins (F = 2.19, df = 5, 192, *p* < 0.05). However, most of the plant families overlapped in the NMDS analysis with a low segregation of plant families in relation to the group of bees visiting their flowers (Figure 6) (Supplementary Material, File 4). The Apiaceae, Asteraceae, Boraginaceae, Brassicaceae and Fabaceae clustered together on the positive side of the second axis, while the Lamiaceae family was on the negative side. *Apis mellifera* contributed negatively to the first axis and was closer to the Brassicaceae than to any of the other plant families. In contrast, mining bees, wild Apidae and Megachilidae, contributed positively to the first component. Wild Apidae and Megachilidae were associated with Lamiaceae, while mining bees were closer to herbaceous plants.

In pan traps, the number of bee genera (x^2^ = 15.2, df = 1, *p* < 0.001) and bee species (x^2^ = 7.22, df = 1, *p* < 0.01) were significantly correlated with the floral resources, but the Shannon index and floral resources were not significantly correlated (x^2^ = 0.001, df = 1, *p* = 0.9752) (Supplementary Material, File 6). In visual sampling, the number of *A. mellifera* registered visiting flowers was positively correlated with the total floral resources within the sampling squares (x^2^ = 54.9, df = 1, *p* < 0.001), in particular, with the floral resources offered by Boraginaceae, Brassicaceae and Lamiaceae, in contrast, a negative correlation was found with Asteraceae (Table 3). Mining bees showed a response similar to that of *A. mellifera*, the number of bees visiting sampling squares being positively correlated with the total floral resources (x^2^ = 8.20, df = 1, *p* < 0.01), and particularly those offered by Boraginaceae and Brassicaceae (Table 2). The abundance of Megachilidae was not correlated with the total floral resources (x^2^ = 0.499, df = 1, *p* = 0.480), but was positively correlated with the floral resources of Lamiaceae (Table 3). For more details about these statistical analyses see Supplementary Material, File 5.

## 4. Discussion

### 4.1. Floral Margins and Foraging Behaviour of Bees

Agricultural landscapes are important areas for the conservation of wild bees and other pollinating insects; however, intensively managed agricultural areas generally offer limited floral resources for the maintenance of bee communities [77]. In the present work, according to our first working hypothesis, the restoration of field margins with flowering herbaceous and shrubby plant species produced an increase in the abundance and diversity of bees, compared to non-restored margins. Several studies have outlined the importance of areas with mixed flowering plants close to field crops in the maintenance of the abundance and diversity of pollinating insects in agricultural landscapes [55,70,74,90,91]. The restoration of field margins with varied flowering species has been among the strategies more frequently adopted for the conservation of wild bees and other pollinating insects in agricultural settings [56,57,66,77,78,92,93,94,95,96,97,98,99,100]. Agro-ecological practices aimed at increasing farmland floral biodiversity have been reported to have an especially high impact on the communities of pollinators in depauperate agricultural landscapes [97,101].

According to our second working hypothesis, qualitative differences were registered in the abundance of most of the groups of bees in relation to the margin type. However, these differences depended largely on the sampling method used. For instance, with the exception of *A. mellifera*, the number of bees captured in pan traps was significantly higher in herbaceous than in shrubby margins for most of the main bee families (i.e., Halictidae, Andrenidae and Megachilidae). In contrast, with visual sampling, a significantly higher number of wild Apidae was found visiting flowers in shrubby than in herbaceous plants. Besides, the number of *A. mellifera* was significantly higher in herbaceous than in shrubby margins. The discrepancies in the estimates of bee abundance between pan traps and visual observations are most likely due to the particularities of the two sampling methods. The efficacy of pan traps is known to vary depending on the abundance of floral resources and bee preference, with low floral resources or low-preferred flowers increasing the attraction of bees to pan traps [46,102,103]. In this case, the low preference of wild Apidae for the plants present in herbaceous margins could have increased their attraction to pan traps, resulting in overestimating their abundances in those margins.

The results of the present work show the importance of the composition of floral margins for the maintenance of bees according to their foraging behaviour. For instance, *Apis mellifera* and mining bees were highly polylectic, having been observed on 17 and 14 plant species, from six plant families, respectively. In agreement with their polylectic habits, the activity of *A. mellifera* and mining bees was found to be positively correlated to the total available floral resources in margins, in particular to the blossoming of Boraginaceae and Brassicaceae (and of Lamiaceae, in the case of *A. mellifera*). The higher abundance of mining bees (namely Andrenidae and Halictidae) in herbaceous margins agrees with the observations of other authors who found that Andrenidae visited mostly herbaceous plants, underlining the importance of annual plants to the survival of this bee family [104]. Potts et al. [79] reported that the presence of andrenids was associated with the overall diversity of all flowers, but especially with that of annuals because most of these have shallow flowers of easy access that can be exploited by short-tongued bees. In contrast, Megachilidae and wild Apidae were more restricted in their foraging habits than *A. mellifera* and mining bees: megachilids were observed on 11 plant species from four families (Boraginaceae, Brassicaceae, Fabaceae and Lamiaceae) but they mainly visited *E. vulgare* and *Ballota hirsuta*; wild Apidae were observed on five plant species from three families (Asteraceae, Fabaceae and Lamiaceae) but most of their visits were registered on two Lamiaceae species (i.e., *B. hirsuta* and *L. stoechas*). The NMDS analyses showed that wild Apidae and Megachilidae were associated with Lamiaceae, the abundance of Megachilidae being highly correlated to the floral resources of this plant family. The results of the present work agree with the observations of other authors in Mediterranean environments. Herrera [12] found that long-tongued Megachilidae, wild Apidae and *A. mellifera* dominated the assemblage of bees associated with *Lavandula latifolia* (Lamiaceae), a plant with a tubular, zygomorphic flower and nectaries at the end of the narrow corolla tube, like most of the Lamiaceae. Petanidou & Ellis [104] also found that annual and perennial plants differed in the type of bees that visited them: annuals were associated with the small, short-tongued Andrenidae, while perennials were associated with large, long-tongued bees such as the Megachilidae and wild Apidae.

Annual variations were registered in the abundance of some groups of bees. A higher number of Halictidae were collected in pan traps in the first year, compared to the second. These variations might be due to normal population cycles, or they might be related to the availability of floral resources. The change in the floral composition of the margins, with the replacement of Asteraceae by Brassicaceae in the second year in herbaceous margins, could be responsible for the variation in the abundance of Halictidae. The higher number of *A. mellifera* in the second than in the first year could have been due to the higher concentration of apiaries in the area in the second year; beekeeping in Spain is mostly nomadic and there are great variations in the abundance of *A. mellifera* among years depending on where beekeepers decide to place their colonies.

### 4.2. Bee Diversity and Conservation

The 2374 specimens captured in pan traps belonged to six families, 18 genera and 58 species. These findings agree with the richness values reported by other authors in agricultural environments. Ortiz-Sánchez and Aguirre-Segura [105,106] reported between 42 and 58 species, belonging to 17 genera and five families, in studies of the Apoidea carried out in semi-natural habitats (i.e., *Medicago* fields surrounded by shrubby xeric vegetation) in the southeast of the Iberian Peninsula. The number of species found in the present work represents approximately 18% and 5% of the species reported for the Andalusian and Iberian bee fauna, respectively [107]. However, it has to be considered that some of the specimens could not be identified to the species level due to their bad state and/or the absence of reliable taxonomic keys or specimens of reference; therefore, it is quite likely that the actual diversity of the study area is indeed higher than that reported here. Nonetheless, these low values are in contrast with the high biodiversity of the Iberian Peninsula, which is one of the hot spots for bee diversity [42,107], and probably reflect the impact of intensive agriculture on the structure of bee communities. Low bee diversity has also been reported for other intensive agricultural habitats, such as cantaloupe fields in France (104 species) and oilseed rape fields in the United Kingdom (26 species) and Germany (27 species) [43]. Other Mediterranean habitats with a lower degree of disturbance, such as phrygana habitats composed of low shrubs and managed olive groves with a wide diversity and high abundance of flowering ruderal plants in the Greek island of Lesvos, showed a higher bee diversity (i.e., 203 and 221 bee species, respectively) [46]. This pattern of bee diversity (262 species) was confirmed by other studies carried out in phrygana ecosystems in mainland Greece [104]. A similar number of bee species (170) was identified in a Mediterranean forest with common understorey plants (e.g., *Cistus salvifolius* L.—Cistaceae, *Salvia fruticosa* Miller—Lamiaceae) regenerating from fire events of different ages, in Israel [79]. Nevertheless, there is a great variation in the number of bee species reported for Mediterranean natural habitats across the world, with figures ranging between 80 and 262 species [104].

Field margins may contribute greatly to the conservation of wild bees. Most of the surface of the Earth is devoted to agriculture [108] and, therefore, strategies aiming to increase the diversity of cropland are expected to have a great impact on the conservation of biodiversity [25,52,77,109]. The numbers of species collected in herbaceous (43) and shrubby (40) margins were much higher than in non-vegetated margins (26). Besides, the percentages of rare species (i.e., representing <1% of the total captures) were higher in shrubby (47.5%) and herbaceous (52.5%) margins than in non-vegetated stands (27.1%). This diversity pattern agrees with other faunistic studies. For instance, in a study carried out in five European countries representing different biogeographical regions, Westphal et al. [43] reported that 32% of the species were singletons or doubletons. Morandin and Kremen [78] registered a greater abundance of uncommon bee species in hedgerow sites of perennial shrubs and grasses native to California’s Central Valley than in weedy unmanaged edges, suggesting that the restoration with perennial native plants was vital to support the diversity of pollinators, especially for the less common species. In the same way, Hannon and Sisk [77] reported that floral resources in hedgerows attracted bee species that were uncommon in the surrounding agricultural landscape. A constant in bee communities is that they seem to be dominated by just a few species. In the present work, we found that five species accounted for 80% of the specimens collected in pan traps. Potts et al. [79] showed that three species accounted for 60% of all the bees in areas of Mediterranean woodland and regenerating post-fire bushy vegetation (phrygana).

Floral margins may contribute to the conservation of threatened or rare species in areas of intensive agriculture. Several species, such as *Andrena flavipes, Andrena humilis, Andrena lepida, Andrena senecionis* and *Andrena tenuistriata*, were more abundant in herbaceous than in shrubby and non-vegetated areas. Three of these species are catalogued as “Of Less Concern” and two as “Data Deficient” in the European Red List of Bees [48]. In contrast, *Panurgus calcaratus* (“Of Less Concern”) and two of the species catalogued as “Nearly Threatened”, *Lasioglossum mandibulare* and *Andrena ovatula*, were more abundant in shrubby than in herbaceous and control margins. These results underline the importance of flowering shrubs in the support of native bees that are uncommon in agricultural landscapes, and of plant diversity for the conservation of bees; vegetated areas in agricultural landscapes may also help to decrease the potential risk of extinction of rare species [53,77]. A lack of data affects most of the world’s bee species, even those of the “well known” European fauna, and represents the biggest problem for the establishment of conservation strategies [20,110]. This situation is especially worrisome in the Iberian Peninsula, which harbours one of the world’s richest bee communities [42,107]. The present work provides information about the abundance of some of the species found in agroecosystems of southern Spain; among them, many rare species (38 out of 58, 64.4%) catalogued as “Of Less Concern”, and ten that are considered as “Data Deficient” in the European Red List of Bees. The low abundance of these species makes one wonder if the Red List reflects the real status of the species, and highlights the need to increase the monitoring effort in order to have proper knowledge of the current status of the species.

## 5. Conclusions

The restoration of field margins with flowering plant species may contribute to the maintenance of bee communities in areas of intensive agriculture. This work is, to our knowledge, the first one using both herbaceous and shrubby plants for the restoration of field margins and measuring the response of wild bees. Until now, most of the works have been carried out in temperate regions using herbaceous plants and have focused on bumble bees. Xerophytic shrubby plants of the Mediterranean area are especially interesting because they are well adapted to the dry environment and, therefore, they need little water and maintenance. Besides, the community of bees associated with shrubby margins differed significantly from the one associated with herbaceous stands, which indicates the need to use a wide range of plant species with different floral traits for the maintenance of rich bee communities and to support rare species. This work provides a well of information on the abundance of many bee species catalogued as “Data Deficient” or “Threatened”, which may help in the establishment of conservation strategies.

## Figures and Tables

**Table 1:** Plant species used in the herbaceous and shrubby plant mixtures. Grams of seeds per square metre (g/m^2^) for herbaceous plants, number of plants per square metre (N/m^2^) for shrubs.

Family	Herbaceous Plants	g/m^2^	Family	Shrubby Plants	N/m^2^
Boraginaceae	*Borago officinalis*	0.50	Fabaceae	*Anthyllis cytisoides*	0.10
Asteraceae	*Calendula officinalis*	1.13	Lamiaeae	*Ballota hirsuta*	0.10
Brassicaceae	*Diplotaxis catholica*	0.10	Fabaceae	*Dorycnium pentaphyllum*	0.10
Apiaceae	*Daucus* sp.	1.00	Fabaceae	*Genista umbellata*	0.15
Boraginaceae	*Echium vulgare*	0.25	Lamiaceae	*Lavandula dentata*	0.25
Fabaceae	*Medicago sativa*	0.63	Lamiaceae	*Lavandula stoechas*	0.25
Fabaceae	*Melilotus officinalis*	0.63	Lamiaceae	*Phlomis purpurea*	0.25
Ranunculaceae	*Nigella damascena*	0.25	Lamiaceae	*Rosmarinus officinalis*	0.10
Lamiaceae	*Salvia verbenaca*	0.50	Lamiaceae	*Salvia officinalis*	0.10
Caryophyllaceae	*Silene vulgaris*	0.50	Asteraceae	*Santolina chamaecyparisus*	0.10
Fabaceae	*Vicia sativa*	0.50	Lamiaceae	*Thymus vulgaris*	0.25

**Table 2:** List of species found in pan traps of shrubby (S), herbaceous (H) and control (C) margins. Red list status (St): DD (Data Deficient), VU (Vulnerable), LC (Least Concern), EN (Endangered), NT (Near Threatened) and CR (Critical Endangered). Tot, total number of individuals captured in pan traps; %, percentage of captures in relation to the total of bees.

Family	Species	St	Margin			Tot	%
			S	H	C		
Andrenidae	*Andrena asperrima* Pérez, 1895	LC	2	1	0	3	0.2
	*Andrena ferrugineicrus* Dours, 1872	LC	0	10	0	10	0.6
	*Andrena flavipes* Panzer, 1799	LC	17	62	10	89	5.7
	*Andrena humilis* Imhoff, 1832	DD	7	16	1	24	1.5
	*Andrena lepida* Schenck, 1861	DD	1	14	0	15	1.0
	*Andrena nigroaenea* (Kirby, 1802)	LC	0	3	0	3	0.2
	*Andrena nilotica* Warncke, 1967	DD	0	1	0	1	0.1
	*Andrena ovatula* (Kirby, 1802)	NT	9	3	0	12	0.8
	*Andrena pilipes* Fabricius, 1781	LC	1	3	1	5	0.3
	*Andrena senecionis* Pérez, 1895	LC	8	14	2	24	1.5
	*Andrena tenuistriata* Pérez, 1895	LC	5	18	1	24	1.5
	*Andrena thoracica* (Fabricius, 1775)	DD	0	1	0	1	0.1
	*Andrena verticalis* Pérez, 1895	LC	0	10	1	11	0.7
	*Panurgus calcaratus* (Scopoli, 1763)	LC	13	3	2	18	1.2
	*Panurgus cephalotes* Latreille, 1811	LC	13	14	6	33	2.1
Apidae	*Amegilla albigena* (Lepeletier, 1841)	LC	1	0	0	1	0.1
	*Amegilla quadrifasciata* (de Villers, 1789)	LC	1	0	0	1	0.1
	*Apis mellifera* Linnaeus, 1758	DD	245	285	230	760	48.7
	*Ceratina cucurbitina* (Rossi, 1792)	LC	0	2	0	2	0.1
	*Ceratina cyanea* (Kirby, 1802)	LC	0	0	1	1	0.1
	*Eucera elongatula* Vachal, 1907	DD	9	10	0	19	1.2
	*Eucera notata* Lepeletier, 1841	DD	81	115	52	248	15.9
Colletidae	*Colletes abeillei* Pérez, 1903	LC	1	0	0	1	0.1
	*Colletes dusmeti* Noskiewicz, 1936	LC	0	1	0	1	0.1
	*Hylaeus taeniolatus* Förster, 1871	LC	0	2	2	4	0.3
	*Hylaeus variegatus* (Fabricius, 1798)	LC	0	1	0	1	0.1
Halictidae	*Halictus fulvipes* (Klug, 1817)	LC	1	1	0	2	0.1
	*Halictus gemmeus* Dours, 1872	LC	1	3	1	5	0.3
	*Halictus subauratus* (Rossi, 1792)	LC	1	1	0	2	0.1
	*Halictus vestitus* Lepeletier, 1841	LC	1	1	1	3	0.2
	*Lasioglossum albocinctum* (Lucas, 1846)	LC	6	1	1	8	0.5
	*Lasioglossum callizonium* (Pérez, 1895)	LC	1	2	0	3	0.2
	*Lasioglossum discus* (Smith, 1853)	LC	1	3	1	5	0.3
	*Lasioglossum interruptum* (Panzer, 1798)	LC	39	51	4	94	6.0
	*Lasioglossum leucozonium* (Schrank, 1781)	LC	4	2	0	6	0.4
	*Lasioglossum malachurum* (Kirby, 1802)	LC	10	34	23	67	4.3
	*Lasioglossum mandibulare* (Morawitz, 1866)	NT	4	1	2	7	0.4
	*Lasioglossum minutissimum* (Kirby, 1802)	LC	0	5	0	5	0.3
	*Lasioglossum parvulum* (Schenck 1853)	LC	3	0	2	5	0.3
	*Lasioglossum pauxillum* (Schenck, 1853)	LC	3	1	5	9	0.6
	*Lasioglossum villosulum* (Kirby, 1802)	LC	2	0	1	3	0.2
	*Lasioglossum virens* (Erichson, 1835)	EN	0	1	1	2	0.1
	*Nomioides minutissimus* (Rossi, 1790)	LC	0	0	1	1	0.1
	*Ceylalictus variegatus* (Olivier, 1789)	LC	0	0	1	1	0.1
Megachilidae	*Anthidium punctatum* Latreille, 1809	LC	0	0	1	1	0.1
	*Anthidium taeniatum* Latreille, 1809	DD	0	1	0	1	0.1
	*Hoplitis acuticornis* (Dufour & Perris, 1840)	LC	1	0	0	1	0.1
	*Hoplitis adunca* (Panzer, 1798)	LC	0	1	0	1	0.1
	*Hoplitis ochraceicornis* (Ferton, 1902)	LC	1	1	0	2	0.1
	*Hoplitis papaveris* (Latreille, 1799)	LC	1	3	0	4	0.3
	*Osmia aurulenta* Panzer, 1799	LC	1	1	0	2	0.1
	*Osmia ferruginea* Latreille, 1811	LC	1	0	0	1	0.1
	*Osmia latreillei* (Spinola, 1806)	LC	1	0	0	1	0.1
	*Osmia tricornis* Latreille, 1811	LC	1	0	0	1	0.1
	*Osmia niveata* (Fabricius 1804)	LC	0	1	0	1	0.1
	*Rhodanthidium infuscatum* (Erichson, 1835)	DD	1	0	0	1	0.1
	*Rhodanthidium sticticum* (Fabricius, 1787)	DD	3	0	0	3	0.2
Melittidae	*Dasypoda cingulata* Erichson, 1835	LC	1	0	0	1	0.1

**Table 3:** Coefficients and statistics of the GLMMs for the analysis of the abundance of different groups of bees in the visual sampling as a function of the floral resources of the different plant families in margins. SE, standard errors of the coefficients; df, degrees of freedom.

Bee Group	Plant Family	Coefficient	SE	x^2^-Value	df	*p*-Value
***A. mellifera***	Apiaceae	−191.1	136.8	2.031	1	0.1541
	Asteraceae	−2.925	1.195	6.234	1	0.0125
	Boraginaceae	3.699	0.343	120.8	1	<0.001
	Brassicaceae	1.987	0.232	76.58	1	<0.001
	Fabaceae	0.593	1.123	0.291	1	0.5899
	Lamiaceae	2.798	0.663	18.54	1	<0.001
**Megachilidae**	Apiaceae	−2.630	2.321	1.336	1	0.2477
	Asteraceae	−3.287	3.445	0.947	1	0.3305
	Boraginaceae	0.701	1.062	0.454	1	0.5004
	Brassicaceae	0.456	0.987	0.222	1	0.6375
	Fabaceae	0.905	1.578	0.342	1	0.5586
	Lamiaceae	2.922	1.521	3.842	1	0.0500
**Mining bees**	Apiaceae	0.831	0.832	1.038	1	0.3083
	Asteraceae	0.581	1.135	0.273	1	0.6014
	Boraginaceae	1.148	0.504	5.395	1	0.0202
	Brassicaceae	0.977	0.444	5.033	1	0.0249
	Fabaceae	0.257	1.547	0.029	1	0.8654
	Lamiaceae	0.707	1.576	0.210	1	0.6471

## Supplementary Materials

The following are available online at https://www.mdpi.com/2075-4450/11/1/26/s1, File 1. Models, procedures and results of the analyses of bee richness (number of genera and species) and diversity (Shannon-Wiener index).File 2. Models, procedures and results of the analyses of the abundance of bees on pan traps.File 3. Models, procedures and results of the analyses of the abundance of bees in visual samplings.File 4. Procedures and results of the Kruskal’s non-metric multidimensional scaling (NMDS) analyses.File 5. Models and results of the analysis of the relationship between bee abundance and floral resources.File 6. Models, procedures and results of the analysis of the relationship between bee richness (Number of genera and species) and diversity *versus* floral resources.
